# Tandem Mass Tag (TMT) Proteomic Analysis of Saliva in Horses with Acute Abdominal Disease

**DOI:** 10.3390/ani11051304

**Published:** 2021-04-30

**Authors:** Alberto Muñoz-Prieto, Damián Escribano, María Dolores Contreras-Aguilar, Anita Horvatić, Nicolas Guillemin, Stine Jacobsen, José Joaquín Cerón, Vladimir Mrljak

**Affiliations:** 1 Clinic for Internal Diseases, Faculty of Veterinary Medicine, University of Zagreb, Heinzelova 55, 10 000 Zagreb, Croatia; alberto.munoz@um.es (A.M.-P.); nicolas.guillemin@hotmail.fr (N.G.); vmrljak@vef.hr (V.M.); 2 Interdisciplinary Laboratory of Clinical Analysis, Interlab-UMU, Regional Campus of International Excellence ‘Campus Mare Nostrum’, University of Murcia, 30100 Murcia, Spain; det20165@um.es (D.E.); mariadolores.contreras@hotmail.com (M.D.C.-A.); 3 Department of Chemistry and Biochemistry, Faculty of Food Technology and Biotechnology, University of Zagreb, Pierottijeva 6, 10 000 Zagreb, Croatia; horvatic.ani@gmail.com; 4 Department of Veterinary Clinical Sciences, Veterinary School of Medicine, Sektion Medicine and Surgery, University of Copenhagen, Hoejbakkegaard Allé 5, DK-2630 Taastrup, Denmark; stj@sund.ku.dk

**Keywords:** tandem mass tag, proteomic, saliva, horses, acute abdominal disease

## Abstract

**Simple Summary:**

This study shows for the first time the variation of the salivary proteome in horses with acute abdominal disease (AAD) compared with healthy horses through a high-throughput proteomic approach. A total of 118 proteins were identified, and 17 showed significant changes between the two groups. The changes observed in proteins were closely related to an impaired primary immune defense and antimicrobial capacity in the mucosa, and one salivary protein (lactoferrin) was successfully verified. These results may increase the background and knowledge of saliva composition in horses with AAD and further understanding of the physiopathological changes occurring in the organism in this disease.

**Abstract:**

The aim of this study was to investigate the changes in the salivary proteome in horses with acute abdominal disease (AAD) using a tandem mass tags (TMT)-based proteomic approach. The saliva samples from eight horses with AAD were compared with six healthy horses in the proteomic study. Additionally, saliva samples from eight horses with AAD and eight controls were used to validate lactoferrin (LF) in saliva. The TMT analysis quantified 118 proteins. Of these, 17 differed significantly between horses with AAD and the healthy controls, 11 being downregulated and 6 upregulated. Our results showed the downregulation of gamma-enteric smooth muscle actin (ACTA2), latherin isoform X1, and LF. These proteins could be closely related to an impaired primary immune defense and antimicrobial capacity in the mucosa. In addition, there was an upregulation of mucin 19 (MUC19) and the serine protease inhibitor Kazal-type 5 (SPINK5) associated with a protective effect during inflammation. The proteins identified in our study could have the potential to be novel biomarkers for diagnosis or monitoring the physiopathology of the disease, especially LF, which decreased in the saliva of horses with AAD and was successfully measured using a commercially available immunoassay.

## 1. Introduction

There is a growing interest in saliva as a biological sample since it can reflect physiological changes in the organism and be a possible source of biomarkers for diagnosing and monitoring diseases [1]. Compared to blood or urine, the main advantage of saliva is its easy and noninvasive collection [2]. In horses, cortisol is probably the most commonly measured analyte in saliva, being used as a biomarker of stress [3]. However, other biomarkers of stress (such as alpha-amylase), immune system (such as adenosine deaminase), or tissue damage (such as creatine kinase) can be analyzed in this fluid [4].

Acute abdominal disease (AAD), also known as colic, is a condition that affects the equine population and is characterized by signs of abdominal pain [5]. The main causes of AAD are intestinal obstruction or strangulation, non-strangulating infarction, enteritis, peritonitis, and ileus [6]. It is one of the most prevalent diseases in horses (it affects up to 10% according to some reports) and a major cause of morbidity and mortality in the species [6]. Some colics respond to medical therapy or spontaneously resolve, while approximately one fifth are complicated cases with obstructions or strangulations that may require intensive medical care and surgical treatment [7]. Breed predisposition has been postulated in some studies where the Arabian breed showed a high prevalence of AAD [8,9,10]. Also, certain breed predilections for specific types of colic have been suggested. For example, fecaliths and impactions of the small colon seem to be more prevalent in younger miniature horses [11,12]. In horses with AAD, various salivary analytes, such as alpha-amylase (sAA), creatine kinase, urea, γ-glutamyl transferase, total bilirubin, total proteins, and phosphorus, showed significant changes compared to healthy horses [13]. In these cases, sAA activity correlated positively with a pain scale, showing potential as a biomarker of prognosis [14,15]. Therefore, saliva is a specimen that contains analytes that can be potential biomarkers of AAD and can help provide knowledge about the physiopathological mechanisms occurring in this disease.

Proteomics can identify high numbers of proteins simultaneously and is therefore useful in identifying new biomarkers as well as individual component proteins and peptides in complex biological samples [16,17]. The use of tandem mass tag (TMT) in proteomics allows the relative simultaneous quantification of differentially labelled peptides, since each peptide is marked and distinguished by differences on its reporter ion masses [18,19]. One study described horses’ salivary proteome with systemic inflammation and found that 57 of 195 unique proteins, detected by liquid chromatography–tandem mass spectrometry, were present in saliva from horses with systemic inflammation but not in saliva from healthy horses [20]. However, to the authors’ knowledge, no studies have investigated the salivary proteome in horses with AAD. 

This study hypothesized that new biologically important proteins could be detected in saliva from horses with AAD by gel-free proteomics using TMT. Therefore, the objective of this report was to analyze the changes in salivary proteomes in AAD, in order to identify new proteins that could provide knowledge about the physiopathological changes that can occur in this fluid and identify new potential biomarkers of this disease.

## 2. Materials and Methods

This study consisted of two parts. First, a proteomic analysis using TMT as a labelling reagent was performed in the saliva of horses with AAD and healthy horses, followed by statistical analysis. Differentially abundant proteins were used to elucidate the pathways affected by AAD. Then, one of the proteins that significantly differed in abundance, lactoferrin (LF), was selected to be validated in the saliva of an additional population of horses with AAD using a commercially available ELISA assay.

### 2.1. Animals

Samples from two groups of horses were analyzed through TMT-based proteomic analysis:

- Diseased horses (*n* = 7; 4 stallions and 3 mares; mean age = 8 years (range 1–18); Andalusians (*n* = 5) and crossbred (*n* = 2)). The presence of AAD was diagnosed based on history, physical examination (abdominal auscultation, rectal examination, and nasogastric intubation), and additional diagnostic tests, including complete blood count (CBC), serum biochemistry profile, abdominal ultrasound, and/or abdominocentesis. Final diagnoses were impaction of ascending colon (*n* = 3), right dorsal colon displacement (*n* = 2), and spasmodic colon (*n* = 2).
- Healthy horses (*n* = 6, 3 geldings, 2 stallions and 1 mare; mean age = 12 years (range 4–15); Andalusians (*n* = 4) and crossbred (*n* = 2)). Horses were found healthy based on history, clinical examination, CBC, and serum biochemistry profile.

These procedures were approved by the ethical committee of the University of Murcia (CEEA 288/2017).

For the validation of the lactoferrin assay, two additional groups were used:

- Diseased horses (*n* = 8; 7 geldings and 2 stallion; mean age = 10 years (range 4–16); Andalusian (*n* = 2), Warmblood (*n* = 2), Holsteiner (*n* = 1) and crossbred (*n* = 3)). Presence of AAD was diagnosed as described above. Final diagnoses were spasmodic colon (*n* = 3), right dorsal colon displacement (*n* = 3), and ascending colon impaction (*n* = 2).
- Healthy horses (*n* = 8; 4 geldings and 4 stallions; mean age = 7 years (range 3–11); Andalusians (*n* = 7) and crossbred (*n* = 1)).

Horses were kept individually in traditional horse stalls, and they were used as dressage or eventing horses. All horses were fed a commercial diet based on oats twice a day. They also had free access to hay and water. All horses followed the standard parasite control measures.

### 2.2. Sampling of Saliva Specimens

Saliva samples were obtained before treatment of the horses by introducing a sponge in the horse’s mouth for 1 min, and thereafter the sponge was placed in a collection device (Salivette, SARSTEDT AG & Co., Nuremberg, Germany). Samples were obtained between 8:00 and 10:00 a.m. and kept at 4 °C until further processing, which took place within 12 h of sample collection. The Salivette tubes were centrifuged at 4000× *g* for 8 min at 4 °C. The supernatant was transferred to 1.5 mL Eppendorf tubes and stored at −80 °C until analysis, which took place within three months after sample collection. A criterion for sample inclusion was the absence of evident blood contamination. 

### 2.3. Liquid Chromatography—Tandem Mass Spectrometry (LC-MS/MS)

From each sample, 35 μg of acetone-precipitated proteins were subjected to reduction, alkylation, and digestion and were labelled using 6-plex TMT reagents according to manufacturer instructions (Thermo Scientific, Waltham, MA, USA) with some modification, as described previously [21]. Protein concentration was determined by the Bradford assay [22]. A pooled sample, generated by mixing equal protein amounts of all 16 samples, was used as an internal standard in all TMT 6-plex experiments. In short, 35 μg of the samples and internal standards were reduced with 200 mM DTT (Sigma-Aldrich, St. Louis, MO, USA), alkylated with 375 mM iodoacetamide (Sigma-Aldrich, St. Louis, MO, USA), and precipitated with ice-cold acetone (VWR, Radnor, PA, USA) overnight. The samples were then centrifuged, and acetone was decanted. The pellets were resuspended with 50 μL of 100 mM TEAB buffer and digested with trypsin (Promega) overnight at 37 °C (2.5 μg of trypsin per 100 μg of protein). TMT reagents were equilibrated at room temperature, resuspended in anhydrous acetonitrile (LC-MS grade, Thermo Scientific, USA), and added to each sample. The labelling reaction was incubated for one hour at room temperature and then quenched by adding 5% hydroxylamine (Thermo Scientific, USA) for 15 min. The samples were then combined at equal amounts, and 5 μg of each mixed sample set was vacuum dried and stored at −20 °C before further LC–MS/MS analysis. The LC–MS/MS analysis was performed on a Dionex UltiMate 3000 RSLCnano flow system (Dionex, Camberley, UK) and a Q Exactive Plus mass spectrometer (Thermo Fisher Scientific, Bremen, Germany) as described previously [23]. For protein identification, a database search against Equus caballus FASTA files downloaded from the NCBI database (4 November 2019, 55006 entries) was performed.

### 2.4. Bioinformatics

A list of significant peptides expressed as a GI number was converted to a list of gene symbols through bioDBnet (https://biodbnet-abcc.ncifcrf.gov, accessed on 1 October 2018). Then, genes from the *Equus caballus* were converted to their orthologous genes in *Homo sapiens* by Biomart from Ensembl (www.ensembl.org, accessed on 1 October 2018).

Using the Cytoscape (v3.6.1) application CluePedia (1.5.2), the original list of significant genes was enriched with their best interactors according to IntAct, Reactome, and STRING–EMBL databases (a maximum of five added interactions per gene). Then, all genes (original and enriched) were submitted to a Gene Ontology analysis using the Cytoscape application ClueGO (v2.5.2) with the following parameters: GO_Biological Process in *Homo sapiens*, evidence codes used = All_without_IEA, GO level from 3 to 12, Kappa score threshold = 0.4, correction method = Bonferroni step down.

The Gene Ontology (GO) terms generated were submitted to a refinement step by Revigo (revigo.irb.hr) to remove redundant terms, define groups of GO terms, and assign a term as leading GO. All information was represented using Cytoscape with a radial layout. Fold change data for original nodes were added as a color gradient. A list of final GO terms was represented with their respective number of genes (original and enriched) inside each other and their associated *p*-value (expressed as −log10) on a histogram.

### 2.5. Lactoferrin Analysis

LF concentrations were measured in the horses’ saliva for validation of proteomic results using a commercial ELISA assay designed for horse LF (Horse Lactoferrin ELISA kit, MBS041152, MyBioSource, San Diego, CA, USA). This assay showed an intra-assay imprecision less than 15% and high linearity (R > 0.97) to the measure of salivary LF after serial dilutions of a saliva sample.

### 2.6. Statistical Analysis

TMT ion-based relative quantification was performed to obtain the fold changes (FC) between groups [24]. Quantified peptides were compared between the AAD (*n* = 7) and the control (*n* = 6) groups using the nonparametric Wilcoxon test after an outlier correction by a Dixon test in each group. Peptides with a *p*-value < 0.05 were considered significantly different between the disease and control groups. For each peptide, FC was calculated as −log2 (meandisease/meancontrol). Statistical analyses were performed using RStudio v1.1.463. R packages used for statistics were readr, outliers, data.table, plotly, and xlsx.

For the validation of LF in saliva, the distribution of data was evaluated using the D’Agostino–Pearson omnibus normality test. Since the data were not normally distributed, the nonparametric Mann–Whitney U (two-way) test was used to compare salivary LF between horses with AAD (*n* = 8) and healthy controls (*n* = 8).

## 3. Results

### 3.1. Proteomic Changes in the Saliva of Horses with Acute Abdominal Disease

A total of 142 proteins were identified in saliva (Supplementary Material, Table S1. Data are available via ProteomeXchange with identifier PXD025241). Of these, 17 differed significantly between horses with AAD and healthy controls, 11 being downregulated, while six were upregulated (Table 1). The proteins that showed the most significant differences (lower *p*-values) between the disease and control groups in the case of those that were downregulated were gamma-enteric smooth muscle actin (ACTA2), latherin isoform X1, and LF precursor. Regarding upregulated proteins, there were mucin 19 (MUC19) and serine protease inhibitor Kazal-type 5 (SPINK5).

### 3.2. Bioinformatics

**Figure 1:**
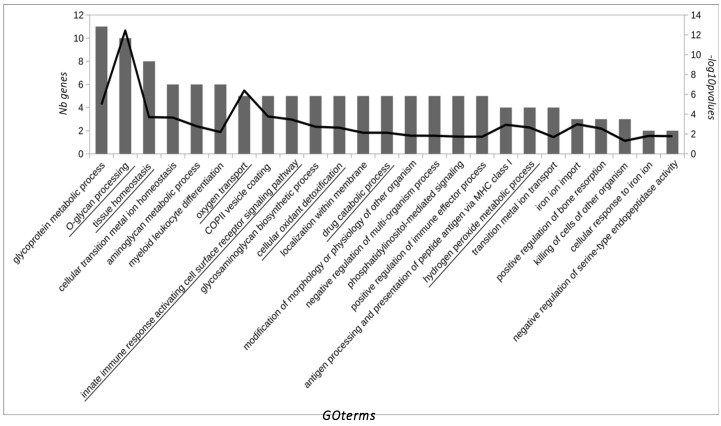
GO terms over-represented in the pool of significantly expressed saliva proteins in horses with AAD. GO terms are ordered by the number of significant genes/proteins of the study associated with them according to the Gene Ontology database (first y-axis). The −log10 of *p*-value for each GO term is represented on the second y-axis. GO terms which define a group of similar GO terms (determined by ReviGO).

**Figure 2:**
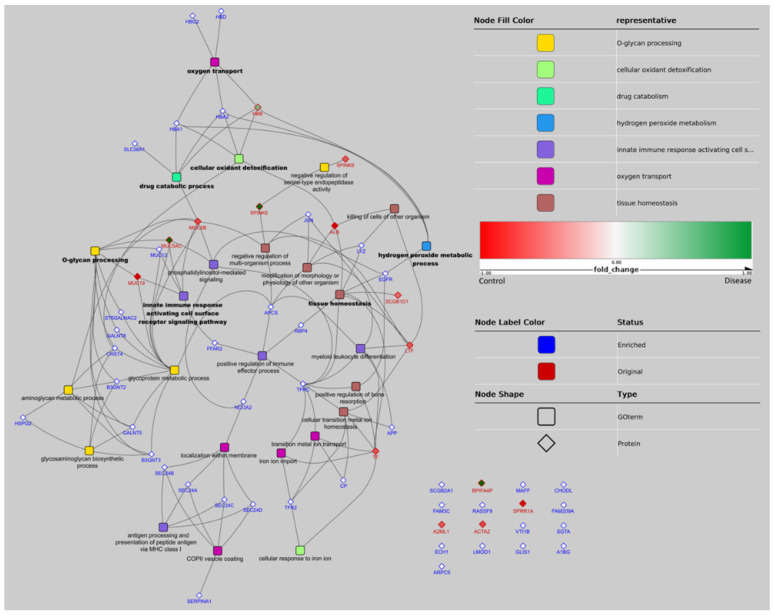
Network representation of GO terms over-represented in the pool of significant proteins and associated proteins. GO terms are represented by a rounded rectangle shape, proteins by a diamond shape. Proteins identified in the experiments have a red border and proteins added by the enrichment step have a blue border. GO nodes are filled by colors corresponding to their GO group (determined by ReviGO). Names of GO terms defining 1 group are in bold. When a protein belongs to 1 GO term, a link is figured between the nodes of the protein and GO term. The network representation has been realized with Cytoscape using the radial layout. Gene acronyms and related full name protein of each protein that appears in the figure (data obtained from UniProt database; (http://www.uniprot.org, accessed on 1 October 2018): (1) Mapped original proteins: ALB (albumin); HBB (hemoglobin subunit beta); LTF (lactoferrin); MUC19 (mucin-19); MUC5AC (mucin-5AC); MUC5B (mucin-5B); SCGB1D1 (secretoglobin family 1D member 1); SPINK5 (serine protease inhibitor Kazal-type 5); SPINK9 (serine protease inhibitor Kazal-type 9); TF (transferrin); (2) Mapped enriched proteins: APCS (serum amyloid P-component); APP (amyloid beta A4 protein); B3GNT2 (N-acetyllactosaminide beta-1,3-N-acetylglucosaminyltransferase 2); B3GNT3 (N-acetyllactosaminide beta-1,3-N-acetylglucosaminyltransferase 3); CHST4 (carbohydrate sulfotransferase 4); CP (ceruloplasmin); EGFR (epidermal growth factor receptor); FFAR2 (free fatty acid receptor 2); GALNT5 (polypeptide N-acetylgalactosaminyltransferase 5); GALNT6 (polypeptide N-acetylgalactosaminyltransferase 6); HBA1 (hemoglobin subunit alpha); HBA2 (hemoglobin subunit alpha; HBD (hemoglobin subunit delta); HBG2 (hemoglobin subunit gamma-2); HSPG2 (basement membrane-specific heparan sulfate proteoglycan core protein); JUN (transcription factor AP-1); LYZ (lysozyme C); MUC12 (mucin-12); NCOA2 (nuclear receptor coactivator 2); RBP4 (retinol-binding protein); SEC24A (protein transport protein Sec24B); SEC24B (protein transport protein Sec24B); SEC24C (protein transport protein Sec24C); SEC24D (protein transport protein Sec24D); SERPINA1 (alpha-1-antitrypsin); SLC38A1 (sodium-coupled neutral amino acid transporter); ST6GALNAC2 (alpha-N-acetylgalactosaminide alpha-2,6-sialyltransferase 2); TFR2 (transferrin receptor protein 2); TFRC (transferrin receptor protein 1); (3) Unmapped original proteins: A2ML1 (alpha-2-macroglobulin-like protein 1); ACTA2 (gamma-enteric smooth muscle-like protein actin); BPIFA4P (putative BPIFA4P protein); SPRR1A (cornifin A); (4) Unmapped enriched proteins: A1BG (alpha-1B-glycoprotein); ARPC5 (actin-related protein 2/3 complex subunit); CHODL (chondrolectin); ECH1 (Delta(3,5)-Delta(2,4)-dienoyl-CoA isomerase, mitochondria); FAM209A (protein FAM209A); FAM3C (protein FAM3C); GLIS1 (zinc finger protein GLIS1); LMOD1 (leiomodin-1); MAFF (transcription factor MafF); RASSF9 (Ras association domain-containing protein 9); SCGB2A1 (secretoglobin family 2A member 1); SGTA (small glutamine-rich tetratricopeptide repeat-containing protein alpha); VTI1B (vesicle transport through interaction with t-SNAREs homolog 1B).

From the 17 proteins identified that showed significant differences, 14 represented unique genes. For the GO analysis, 42 related proteins to these 14 were added in the enrichment step. As a result of the GO analysis, seven GO groups were defined as leader representative groups in AAD in horses: O-glycan processing (10 genes, −log10 *p* = 12.4), tissue homeostasis (eight genes, −log10 *p* = 3.7), oxygen transport (five genes, −log10 *p* = 6.3), innate immune response activating cell surface receptor signaling pathway (five genes, −log10 *p* = 3.4), cellular oxidant detoxification (five genes, −log10 *p* = 2.6), drug catabolic process (five genes, −log10 *p* = 2.1), and hydrogen peroxide metabolic process (four genes, −log10 *p* = 2.6) (Figure 1). The most relevant pathways in our study are highlighted in bold (Figure 2).

### 3.3. Changes in Salivary Lactoferrin Concentration in Horses with Acute Abdominal Disease Measured with the ELISA Assay

**Figure 3:**
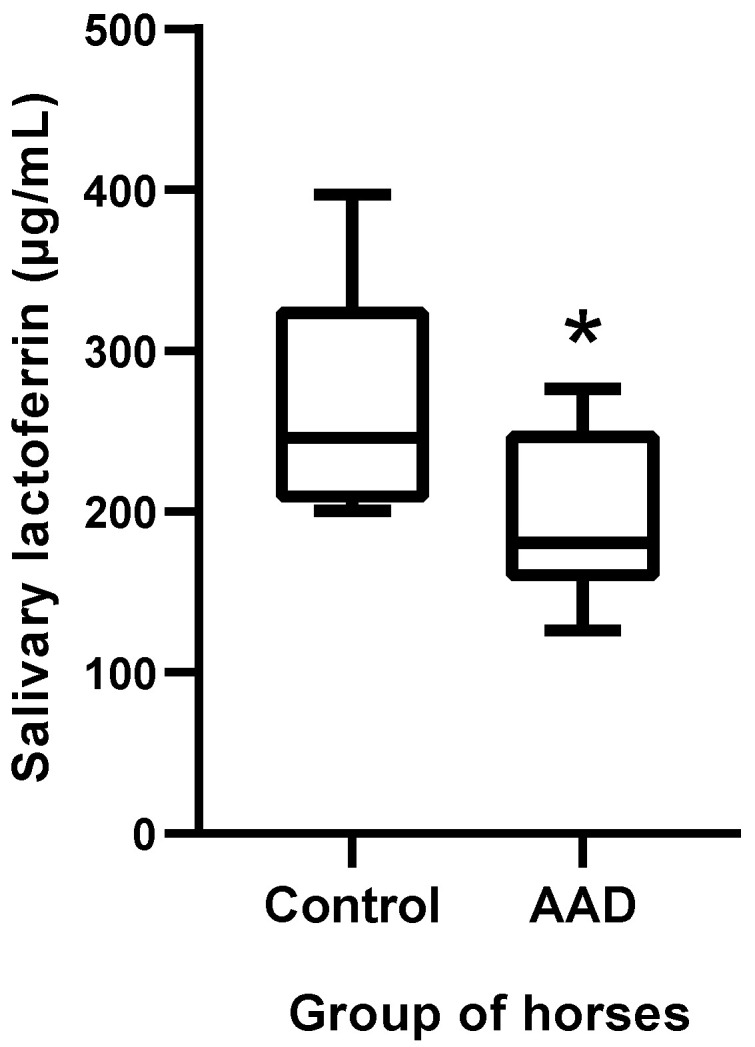
Lactoferrin concentrations in horses with AAD (*n* = 8) compared with healthy horses (*n* = 8). The plot shows the median (line within box), 25th and 75th percentiles (box), and 10th and 90th percentiles (whiskers). Asterisk indicates a significant difference between groups: * *p* < 0.05. AAD: acute abdominal disease.

The salivary concentrations of LF were significantly lower in horses with AAD (median 180.6, range 126.1–276.6 µg/mL) compared with a control group of healthy horses (median 246.1, range 200.6–396.8 µg/mL) (*p* = 0.019) (Figure 3).

## 4. Discussion

The use of saliva has gained interest in the veterinary field in recent years since it can be obtained noninvasively and without stress, and it is very useful for large population studies and serial sampling. It has been used in the evaluation of stress through the determination of cortisol, as well as for the assessment of proteins related to the immune system, such us C-reactive protein, haptoglobin, and immunoglobulins [25]. In this report, we have detected that 17 proteins are differentially abundant in the salivary proteome of horses with AAD compared with healthy ones. Within the most downregulated proteins in horses with AAD, there were ACTA2, latherin isoform X1, and LF precursor, while the most upregulated were MUC19 and SPIKN5. In addition, GO analysis showed that three leader pathways were altered by proteins that change in the saliva of horses with AAD, namely o-glycan processing, innate immune response activating cell surface receptor signaling pathway, and tissue homeostasis.

ACTA2 is part of the cytoskeleton of the intestinal smooth muscles, being involved in their motility [26]. Further studies would be needed to elucidate the reason for the decrease of ACTA2 in the saliva of horses with AAD found in our study. It could be related to gastrointestinal tract inflammation, which, in humans, has been associated with the reduced expression of ACTA2, making this protein a possible clinical marker of intestinal muscle damage [27].

Latherin is a surfactant protein that has been previously detected in the saliva of horses, where it is believed that it helps to enhance the wetting, softening, and maceration of the dry, fibrous food for which equines are adapted [28]. This protein can also have a role in the function of innate immunity at mucosal surfaces [29].

In our study, a commercially available immunoassay kit confirmed the decrease in LF found in horses with AAD by proteomics. To the best of our knowledge, LF has not been previously measured in the saliva of horses. LF is a glycoprotein contained in most mammalian exocrine secretions, including milk, tears, and saliva [30]. It is involved in the primary innate immune defense system of mammals due to its antimicrobial and antiviral activity [31,32,33,34] and has immunomodulatory properties [35]. It is also involved in the immune cell responses through the activation of natural killers and the recruitment of T lymphocytes, monocytes, and neutrophils [36]. In horses, human recombinant LF was shown effective in cases of prolonged inflammation caused by persistent breeding-induced endometritis in mares when it was infused at uterine levels, modulating the presence of PMNs [37]. Also, LF is related to the gastrointestinal tract since it can downmodulate both humoral and cellular components of immunity and have a key role in maintaining gut homeostasis [35]. In this line, LF serum concentration has been used as an indicator of severity in gastrointestinal tract inflammation in various species, including humans, as well as murine and rabbit models [38]. The connection of LF with the tissue homeostasis GO terms detected in our study may confirm the importance of this protein in the maintenance of normal tissue metabolism and function. It is important to point out that stress could also influence this protein since its decrease has been reported in the saliva of sheep after a stressful situation [39].

In this study, we found the upregulation of the serine protease inhibitor Kazal-type 5 (SPINK5), a member of the gene family serine protease inhibitor Kazal-type cluster, which encodes inhibitors of the serine proteases [40]. In line with our results, it was also previously reported in horses’ saliva with inflammation [20], and it could be postulated that this protein could be involved in the regulation of inflammation. Therefore, its increase in horses with AAD could be related to a protective response that the organism produces against this condition.

Our results showed that two mucins were upregulated (MUC19 and MUC5B), and one was downregulated (MUC5AC). Originally, mucins were related to the protection of epithelia by binding cellular debris, bacteria, and viral pathogens [41]. This is in agreement with our results where MUC5AC were closely related to the innate immune response GO terms. The variations in the sequence of glycosylation of mucins generate a variety of epitopes that can be used to distinguish between normal and disease states [42]. This applicability has been widely studied to understand the role of mucins in cancer biology, where they are considered to be promising markers [42]. To the best of the authors’ knowledge, MUC5AC has not been reported in horses’ saliva. However, in humans and mice, it was attributed to a function in the innate and adaptive immune response against bacterial invasion [43]. Furthermore, the upregulation of salivary MUC19 in humans has been postulated as an important factor in the protection of oral health since altered expression of this protein may influence the oral and intestinal microbiota and, therefore, the immune homeostasis [44].

A limitation of this study is that although it is known that AAD is more prevalent in old, rather than young, horses, the effects of age, breed, and gender were not considered. Therefore, the lack of homogeneity of groups may influence the results obtained in our report. In addition, further studies in which LF would be analyzed in a larger population of horses with AAD and compared with healthy horses and horses with other diseases would be recommended in order to determine the diagnostic potential and applications of this biomarker. These studies should evaluate the potential use of LF as part of a biomarker panel in combination with other already established biomarkers, such as lactate, WBC, or SAA. It would also be of interest to investigate the levels of LF in horses with AAD with varying degrees of intestinal compromise (e.g., obstipations, versus strangulations, versus inflammatory/infectious gastrointestinal tract disease). In addition, it would be interesting to evaluate the use of LF as a possible prognostic tool or to assess the response to treatment.

## 5. Conclusions

Seventeen proteins were significantly altered in horses with AAD, with the most downregulated (ACTA2, latherin isoform X1, and LF) being closely related to an impaired primary immune defense and antimicrobial capacity in the mucosa. The most upregulated MUC19 and SPINK5 can be associated with a protective effect during inflammation. The proteins identified in our study using a TMT-based approach could have the potential to be novel biomarkers for diagnosis or monitoring this disease. One of the most promising could be LF, which decreased in the saliva of horses with AAD and can be measured by the use of a commercially available immunoassay.

## Figures and Tables

**Table 1:** Statistically significant expression changes of proteins in horses’ saliva with acute abdominal disease (*n* = 7) in relation to healthy horses (*n* = 6).

Protein Accession ID	Protein Name	*p*-Value	Mean Control	Mean Disease	Fold Change	Regulation in AAD
335773136	Actin, gamma-enteric smooth muscle	0.009	1.83	0.93	0.98	Down
953883212	Latherin isoform X1	0.01	1.4	0.75	0.9	Down
255653068	Lactotransferrin precursor	0.01	1.25	0.97	0.37	Down
255683515	Hemoglobin subunit beta	0.01	1.17	1.09	0.1	Down
545181443	Secretoglobin family 1D member	0.015	2.31	1.02	1.18	Down
349602714	Alpha-2-macroglobulin	0.024	2.02	1.08	0.90	Down
338713575	Serine protease inhibitor Kazal-type 9	0.024	1.67	1.08	0.63	Down
3581959	Lactoferrin, partial	0.024	1.25	0.97	0.37	Down
953869413	Mucin-5AC	0.030	1.26	1.1	0.20	Down
953851776	Polymeric immunoglobulin receptor isoform X1	0.032	1.73	1.54	0.17	Down
953857134	Mucin-19	0.009	1.15	1.76	−0.61	Up
953872813	Serine protease inhibitor Kazal-type 5 isoform X2	0.010	1.06	1.89	−0.83	Up
14456405	Albumin, partial	0.015	0.94	1.51	−0.68	Up
953869415	Mucin-5B	0.026	1.15	1.31	−0.19	Up
953891633	Odorant-binding protein	0.030	1.07	1.35	−0.34	Up
953853589	Cornifin	0.035	0.99	1.17	−0.24	Up
3892523	Transferrin, partial	0.038	0.67	0.7	−0.06	Up

## Data Availability

Proteomic data are available via ProteomeXchange with identifier PXD025241.

## Supplementary Materials

The following is available online at https://www.mdpi.com/article/10.3390/ani11051304/s1, Table S1: TMT proteins list.
